# Incidence and Demographic Patterns of Orofacial Clefts in Mysuru, Karnataka, India: A Hospital-based Study

**DOI:** 10.5005/jp-journals-10005-1542

**Published:** 2018-10-01

**Authors:** Praveen Kumar PS, Kanika S Dhull, Lakshmikantha G, Nikita Singh

**Affiliations:** 1 Associate Professor, Department of Dentistry, Mysore Medical College and Research Institute, Mysuru, Karnataka, India; 2 Reader, Department of Pedodontics and Preventive Dentistry, Kalinga Institute of Dental Sciences, KIIT University, Odisha, India; 3 Assistant Professor, Department of Obstetrics and Gynaecology, Mysore Medical College and Research Institute, Mysuru, Karnataka, India; 4 Postgraduate Student, Department of Obstetrics and Gynaecology, Mysore Medical College and Research Institute, Mysuru, Karnataka, India

**Keywords:** Cleft lip and palate, Congenital, Incidence, Syndrome.

## Abstract

**Objective:**

The study was conducted to know the incidence and trends of orofacial clefts between 1st January 2011 and 31st August 2016 in Mysuru, Karnataka; and to describe the demographic patterns.

**Materials and methods:**

This is a retrospective, hospital-based study. Data were retrieved from multiple sources like Parturition books, Neonatal intensive care unit records, baby and mothers case records archived in medical records department of Cheluvamba Hospital, a Tertiary Care Government Hospital, attached to Mysore Medical College and Research Institute, Mysore, Karnataka, between 1 January 2011 and 31 August 2016. All subjects with CL ± P were included in the study. Data were collected using semi-structured proforma, designed based on the review of the literature. Prior to the study, approval of the Institutional Ethics Committee was obtained. Collected data were subjected to descriptive statistical analysis using SPSS version 21 (SPSS Inc.).

**Results:**

A total of 59 children with CL ± P were born between 1 January 2011 and 31 August 2016 among 77667 births (Male: 36,625; Females: 41042). Hence the incidence of orofacial clefts in this hospital was 0.76/1000 births/year. Incidence in boys was 0.71/1000 births and in girls it was 0.78/1000 births (p < 0.7). Distribution of CL ± P showed that cleft lip with palate were more prevalent, which was 64.4%. 54.2% of the female child had clefts. Pierre-Robin syndrome was the most common associated malformation.

**Conclusion:**

This study indicates that CL ± P are the most common types of orofacial clefts. Adequate ante-natal history in birth records is not been given critical importance, with lack of parental counseling. Public awareness regarding the early diagnosis of orofacial clefts, follow up, surgery and dental therapy is required.

**How to cite this article:** Kumar PSP, Dhull KS, Lakshmikantha G, Singh N, Incidence and Demographic Patterns of Orofacial Clefts in Mysuru, Karnataka, India: A Hospital-based Study. Int J Clin Pediatr Dent, 2018;11(5):371-374.

## INTRODUCTION

Cleft lip with or without palate (CL ± P) is the most frequently occurring congenital malformations of the head and neck among live births, accounting for 65% of all head and neck anomalies. These clefts occur when the lip or palate fail to fuse during their prenatal development (first trimester).^1^ Although hereditary, Cleft Lip-Palate is believed to be multifactorial rather than a single-gene disease with various etiologies like chemical exposures, radiation, maternal hypoxia, teratogenic drugs, nutritional deficiencies, etc., playing a role.^2^ Patients affected with orocial clefts present complex biologic, sociologic and psychologic problems and rehabilitation requires a mul-tidisciplinary approach. The functional outcome depends on the timing of surgery, type of repair, physiotherapy and proper follow-up. Studies have shown that the incidence and prevalence of Cleft lip-palate are 1 in 600 (1:600) and 9.92 per 10,000 worldwide respectively. The Cleft lip and cleft lip-palate prevalence are 3.28 per 10,000, and 6.64 per 10,000.^3^ Cleft lip and palate is more frequently observed in males than females, but the prevalence varies with geographic locations, races, and ethnic groups. Cleft lip-palate can be unilateral or bilateral, may occur as both cleft lip and palate or either on its own. In case of pre alveolar clefts, unilateral clefts (75%) are more frequent than the bilateral clefts (25%). Fifty percent of deformities are combined clefts of the lip and palate. About 25% are bilateral ones. Approximately 30% of cleft lip-palate cases are observed to be associated with a syndrome and are presented with some other anomalies that behave differently epidemio-logically from clefts without associated malformations.^4,5^ Various epidemiological studies conducted worldwide have shown a wide disparity in the risk of developing clefts within races and incidence. Hence this study was conducted to know the incidence and trends of orofacial clefts between 1st January 2011 and 31st August 2016 in Mysore, South India; and to describe the demographic patterns.

## MATERIALS AND METHODS

This is a retrospective, hospital-based study. The data was retrieved from multiple sources like Parturition books, neonatal intensive care unit (NICU) records, baby and mothers case records archived in Medical Records Department of Cheluvamba Hospital, attached to Mysore Medical College and Research Institute, Mysore, Karnataka, between 1 January 2011 and 31 August 2016. All subjects with CL ± P were included in the study. This hospital is a Tertiary Health Care Center run by the government, for women and child with approximately 1300 deliveries done every month, one of the highest in the state. It receives patients from all over Mysore district, including from surrounding districts of south Karna-taka and different strata of society. The characteristics of this hospital made it appropriate for conducting this study. The data was collected using semi-structured proforma, designed based on the review of the literature. Prior to the study, approval of the Institutional Ethics Committee was obtained Collected data were subjected to descriptive statistical analysis using SPSS version 21 (SPSS Inc.). The cleft samples were compared for variables such as types of the cleft, syndrome or non-syndrome, treatment provided, gender, mother’s age, parity, consanguinity, infant’s weight, term. Then, the factors mentioned above were analyzed with Chi-square test.

## RESULTS

A total of 59 children with CL ± P were born between 1 January 2011 and 31 August 2016 among 77667 births (Male: 36,625; Females: 41042). Hence the incidence of orofacial clefts in this hospital was 0.76/1000 births/year. Incidence in boys was 0.71/1000 births and in girls it was 0.78/1000 births (p < 0.7). Distribution of CL ± P (Table 1) shows that cleft lip with palate is more prevalent, which was 64.4%. Least was cleft palate only with 11.9%. 54.2% of the female child had clefts, and male child with clefts was 45.8% (Table 2).

A total of 91.5% of children with clefts were born of mothers, whose age was less than 30 years. 81.4% of births were of 1 or 2 mother’s parity. 59.3% of children’s weight at birth was below 2500 gms. 33.9% were born of the consanguineous marriage. 13.9% of clefts were associated with various syndromes. No treatment was done in the hospital and were referred to other centers, with suitable facilities (Table 3). Pierre-Robin Syndrome was the most common associated malformation (Table 4).

## DISCUSSION

This study was conducted in a tertiary health care hospital in the urban area which receives a large rural population in and around Mysore, and not presenting any significant variation.

There are more than 10 million people having clefts of lip and palate worldwide. The reported incidence is 0.8-1.^6^ for every 1000 births.6 The present study showed the incidence of orofacial clefts in this hospital to be 0.76 / 1000 births/year which is comparable to different studies conducted all over the world. The study conducted in Tehran by Jamilian et al. showed the incidence of CL ± P infants as 2.14 per 1000 live births.^7^ The incidence of CL+ P was 1.81 per 1000 live births in Korea^8^ and 1.91 per 1000 live births in Pakistan.^9^ There is a greater incidence of palatal clefts, with figures of up to 1:300 live births among Native Americans.^10^ Owens et al.^11^ found the incidence rate of 1.4:1000 births in England. In Italy, Calzolari et al.^12^ found 1.3:1000 and 0.6-0.7:1000 live births in Sweden.^13^ In Ireland, the incidence was 1.28:1000.^14^ In Mangalore, India Supriya et al. found the incidence to be 2.42/1000 births/year.^15^

**Table 1:** **Table 1:** Distribution of cleft types

*Type of cleft*		*Total*		*Percent (%)*		*Prevalence in 1000 live births*	
Cleft lip		14		23.7		0.18	
Cleft lip + palate		38		64.4		0.49	
Cleft palate		07		11.9		0.09	
Total		100		100		0.76	

**Table 2:** **Table 2:** Distribution of cleft according to gender

*Type of cleft*		*Girls*		*Boys*		*Total (Percent)*	
Cleft lip		04 (6.8%)		10 (16.9%)		14 (23.7)	
Cleft lip + palate		22 (37.2%)		16 (27.2%)		38 (64.4)	
Cleft palate		04 (6.8%)		03 (5.1%)		07 (11.9)	
Total		30 (50.9%)		29 (49.1%)		59 (100)	

**Table 3:** **Table 3:** Distribution of subjects according to related factors

*Related factors*		*Count (Percentage)*	
Gender			
Girl		32 (54.2)	
Boy		27 (45.8)	
Mother’s age			
Less than 30 years		54 (91.5)	
30 and above		05 (8.5)	
Mother’s Parity			
1 and 2		48 (81.4)	
3 and higher		11 (18.6)	
Consanguineous marriage			
No		39 (66.1)	
Yes		20 (33.9)	
Infant’s Weight			
Above 2500 g		24 (40.7)	
Below 2500 g		35 (59.3)	
Term			
Pre-term		13 (22)	
Term		46 (76)	
Syndrome			
No		51 (86.4)	
Yes		08 (13.6)	
Treatment done			
No		59 (100)	
Yes		00 (0)	
Religion			
Hindu		53 (89.8)	
Muslim		06 (10.2)	

**Table 4:** **Table 4:** Associated malformation

		*Count*		*Percentage*	
*Associated_Malformation*		16		27.1	
Piere-Robin syndrome		3		5.1	
HELLP Syndrome		1		1.7	
Dandy-Walker cyst, Meningocele, Occipital Encephelocoele, multipal neural defects		1		1.7	
Hydrocele with spinabifide with meningocele		1		1.7	
Hypertelorism with depressed Nasal Bridge		1		1.7	
Dysplastic ears, bilateral renal agenesis		1		1.7	
Birth Asphysia, H I E of New Born		1		1.7	
Post-natal poly dactaly		1		1.7	
Hypo spadiasis		1		1.7	
Anaemic		1		1.7	
Preauricular Tag’s		1		1.7	
Broncho pneumonia		1		1.7	
Sirenomelia, DEAD		1		1.7	
Pneumonia, DEAD		1		1.7	

0.76 / 1000 incidence overall

0.71 / 1000 incidence in males

0.79 / 1000 incidence in females

p value = 0.7, Chi-square test

In this study, 54.2% of the female child had clefts, and male child with clefts was 45.8%, which is in contrast to worldwide sex distribution data (Female: Male = 60:40).^16,17^ It was also noted that isolated cleft palate was more common in females. Similar findings were reported by Uppal et al.,^18^ Ibrahim et al.,^19^ Habib.^20^ Studies by Jamilian et al. and Iregbulem^21^ reported that both types of clefts were equally distributed between males and females. A study conducted by Murthy et al.^22^ showed sex distribution of oral clefts was more in males than females. Studies from Europe and the USA^23,24^ showed females were less often affected. A study conducted by Sah and Powar showed among all cleft cases, 55.7% were males, and 44.3% were females.^25^

Sixty-four percent of patients had combined cleft lip and palate, followed by 23.7% with cleft lip and 11.9% with cleft palate. Unilateral clefts are more common than bilateral cleft which were seen only in 5 patients (8.5%). Similar results were shown in the studies conducted by Uppal et al.^18^ and Samuel et al.^26^

In this study, all the patients belonged to the lower socio-economic status. The mother’s age and parity were found not statistically significant in relation to the orofacial clefts. Habib^20^ stated that incidence of the clefts probably increases with mother’s age.

In this study, the association between consanguinity and orofacial clefts was not statistically significant. But 33.9% of patients were born of the consanguineous marriage. Studies by Jamilian^7^ Sah, Powar^25^ Harville^27^ have revealed that the risk of orofacial clefts increased in consanguineous marriage.

In this study, Infants weight of 59.3% of the patients with orofacial clefts was below 2500 gms (Ranging from 1200 gms–2400 gms), which was significant. Studies by Rintala and Gylling,^28^ Jamilian^7^ have shown a relation between the lower average birth weight of infants and orofacial clefts. However, Hennrikson^29^ reported a mean birth weight of 3405.6 gms for children with clefts.

Due to lack of adequate information’s from birth records and ante-natal history, all possible risk factors for every patient was unable to record and assess. If complete ante-natal history is recorded, accurate knowledge of the role of risk factors such as Consanguinity, familial occurrence, Low birth weight, Mothers age, and parity could be assessed.

## CONCLUSION

A total of 59 children with CL ± P were born between 1 January 2011 and 31 August 2016. The incidence of orofacial clefts was 0.76/1000 births/year. Parental counseling was lacking, which necessitates the setting up of cleft clinics at government hospitals. People should be advised to avoid consanguineous marriage. The government should develop adequate infrastructure in the health sector for awareness, identification, and treatment of clefts. Adequate ante-natal history in the birth records need to be accurately recorded. Identification of the risk factors causing clefts is crucial for the prevention and treatment of orofacial clefts. Along with post-surgical care, psychological treatment in the form of parental counseling forms an important aspect of the treatment modality.
